# Complex Retrograde CTO-PCI With Laser Atherectomy and a Novel Tip-In Technique Into a Guide Catheter Extension

**DOI:** 10.1016/j.jaccas.2026.107651

**Published:** 2026-03-28

**Authors:** Fatemeh Mohammed, Manyoo A. Agarwal, Ronney Shantouf, Ashraf Alazzoni

**Affiliations:** Heart Vascular Thoracic Institute, Cleveland Clinic Abu Dhabi, Abu Dhabi, United Arab Emirates

**Keywords:** complex-PCI, laser atherectomy, NSTEMI, TrapLiner

## Abstract

**Objective:**

We present a challenging case of retrograde chronic total occlusion (CTO) percutaneous coronary intervention (PCI) using laser atherectomy and a novel tip-in microcatheter technique in conjunction with a TrapLiner guide extension catheter (Teleflex).

**Case Summary:**

Left heart catheterization showed multivessel disease. The patient was turned down for surgical intervention and hence underwent PCI to the mid right coronary artery and staged retrograde CTO-PCI of the proximal left anterior descending artery. The CTO-PCI procedure was performed using a retrograde approach but because the microcatheter advanced over the externalized wire could not be used to wire into the ongoing left anterior descending artery, we explored innovative ideas. The distal small septal channel was crossed using a tip-in technique wherein the distal tip of an antegrade microcatheter was trapped in a TrapLiner catheter and intubated with the retrograde guidewire. The antegrade microcatheter was advanced over the retrograde wire, and laser atherectomy was performed followed by PCI, with excellent results.

**Conclusions:**

Using advanced techniques such as tip-in maneuvers with equipment such as the TrapLiner can make the difference in complex CTO-PCI cases that otherwise would fail.

## Graphical Abstract

**Figure:**
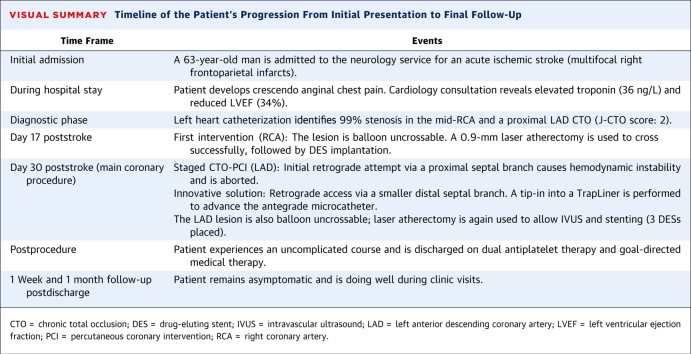

Chronic total occlusions (CTOs) are among the most challenging lesions to treat, often requiring sophisticated techniques and specialized equipment to achieve successful revascularization. The retrograde approach, although associated with higher complication rates compared with the antegrade approach, has been instrumental in improving success rates for complex CTOs. In this report, we describe a complex retrograde CTO percutaneous coronary intervention (PCI) with laser atherectomy and a novel tip-in technique into a TrapLiner guide extension catheter. The technique represents a significant advancement in interventional cardiology, offering improved success rates and enhanced safety profiles for patients with challenging coronary lesions.^1^^,^^2^

**Take-Home Messages**

• Using a TrapLiner during retrograde CTO-PCI provides operators with more guide support and makes it easier to trap a retrograde wire when a microcatheter is used inside a TrapLiner catheter with the trapping balloon inflated.
• This case also highlights the utility of using laser atherectomy for retrograde CTO-PCI.

## Case Summary

**Figure 1:**
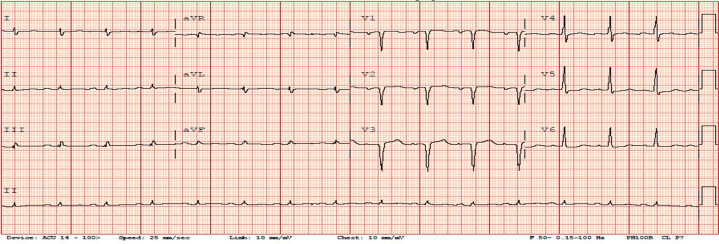
Electrocardiogram Showing Normal Sinus Rhythm With No Significant ST-T Changes

**Figure 2:**
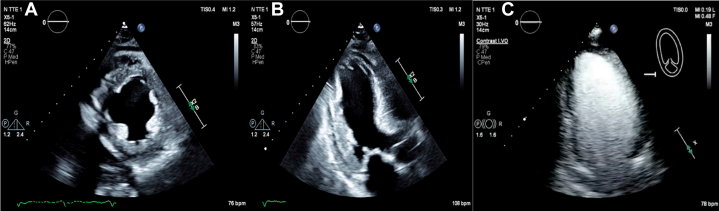
Transthoracic Echocardiogram Showing Reduced Left Ventricular Systolic Function With Regional Wall Motion Abnormality in the Left Anterior Descending Artery Territory

A 63-year-old man known to have type 2 diabetes mellitus on metformin-sitagliptin, hypertension on amlodipine-valsartan, and dyslipidemia on atorvastatin was admitted to the neurology service for acute ischemic stroke confirmed by brain magnetic resonance imaging, which revealed multifocal acute infarcts in the cortical and subcortical right frontoparietal lobe. During his hospital stay, he developed crescendo anginal chest pain, for which the cardiology team was consulted. Electrocardiogram showed sinus rhythm with no significant ST-T changes (Figure 1), and high-sensitivity troponin level was mildly elevated at 36 ng/L. Transthoracic echocardiography revealed reduced left ventricular ejection fraction (34%) with regionality in the left anterior descending artery (LAD) territory and no significant valvular disease (Figure 2).

**Figure 3:**
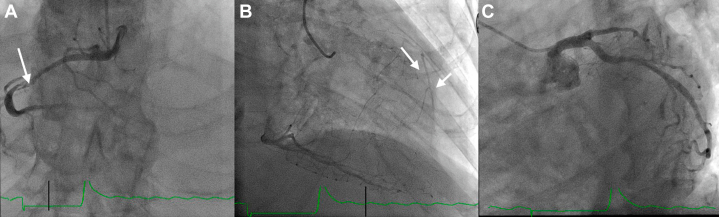
Findings on Investigative Left Heart Catheterization Left heart catheterization showed (A) severe complex mid right coronary artery stenosis (99%; arrow) (B) with robust collaterals to the left coronary system (arrows) and (C) chronic total occlusion of the proximal left anterior descending artery with blunt stump.

Left heart catheterization showed severe complex stenosis in the mid right coronary artery (RCA) and CTO of the proximal LAD with ambiguous cap, with right to left collaterals (Figure 3). During a multidisciplinary heart team discussion, the patient was assessed as being at very high surgical risk, and a joint decision was made to proceed with complex multivessel PCI. His J-CTO (Multicenter CTO Registry of Japan) score was 2, and his CHIP (complex high-risk indicated PCI) score was 6. At 17 days poststroke and with clearance from the neurology team, we proceeded with complex PCI, as the patient was still symptomatic despite being on antianginal medications. He underwent complete revascularization in a staged fashion.

## Procedural Steps

### Access

• During initial PCI: The right radial artery was engaged using an AL 0.75 guide catheter (Asahi).
• During planned staged PCI: The RCA was engaged using an AL1 guide catheter (Asahi) through the right radial artery, and the left main coronary artery was engaged using a PB 3.0 guide catheter (Asahi) through the femoral artery.

### Intervention of the RCA

**Figure 4:**
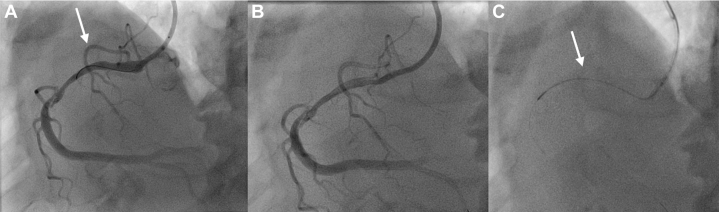
Right Coronary Artery Percutaneous Coronary Intervention Initial Steps Left heart catheterization showing (A) Runthrough wire crossed with FineCross catheter support. (B) Balloon uncrossable lesion. (C) Corsair Pro XS catheter unable to cross. The arrow in A points at Runthrough wire. Arrow in C points at Corsair Pro XS microcatheter.

**Figure 5:**
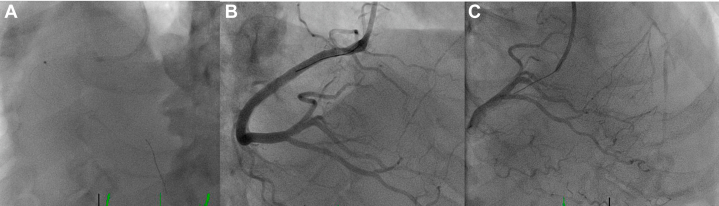
Right Coronary Artery Percutaneous Coronary Intervention Final Steps Left heart catheterization showing (A) 0.9-mm laser atherectomy performed without difficulty. (B) The right coronary artery lesion was predilated with an NC balloon, and percutaneous coronary intervention was performed using a 4.0 × 48 mm drug-eluting stent. (C) Final image of the right coronary artery highlighting the collaterals to the left anterior descending artery.

For the RCA, we used a Runthrough wire to cross the lesion; however, we were unable to cross with a FineCross microguide catheter, small-size balloons, or a Corsair Pro XS microguide catheter. A 0.9-mm laser atherectomy system was eventually able to cross the lesion successfully (Figure 4). The lesion was predilated with an NC (noncompliant) balloon, followed by intravascular ultrasound for stent sizing and assessing for possible complications. PCI to the mid-RCA was performed with a 4.0 × 48 mm Everolimus drug-eluting stent (DES), followed by postdilation with 4.5-mm and 5.0-mm NC balloons (Figure 5). For all manufacturer details, see the Equipment List.

### Intervention of the CTO lesion

**Figure 6:**
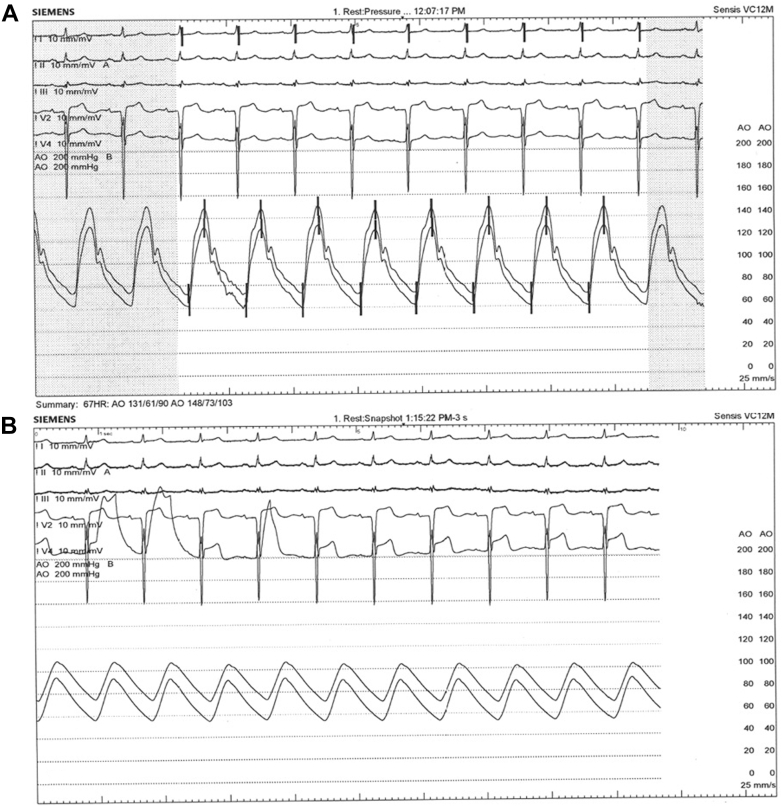
Electrocardiogram and Hemodynamic Tracings in the Catheterization Laboratory Electrocardiogram and hemodynamics (A) at the beginning of the procedure and (B) at the time of crossing the proximal septal branch, when the patient became unstable with decreased blood pressure and ST-segment elevations anteriorly on electrocardiogram.

Given the persistence of his symptoms, the patient then underwent staged PCI to the LAD lesion on day 30 poststroke, with dual access. Dual TrapLiner catheters were used, and we opted for a retrograde approach through a proximal large septal branch. We advanced a Suoh 03 wire into the proximal septal branch (largest branch) through the RCA and followed the wire in retrograde fashion with a Caravel microcatheter. Then, we used a Gaia Next 2 wire to cross into the left main coronary artery, and we confirmed its position on multiple views. We externalized an RG3 wire, and over the externalized wire advanced a microcatheter and attempted to wire the LAD, but the microcatheter was pointing to the septal branch given the proximity of the LAD occlusion and the proximal septal branch. At this time, the patient experienced anginal chest pain along with ST-segment elevations on the monitor, and there was a drop in blood pressure (Figure 6). While this can be an expected adverse effect of using collaterals, it forced us to change plans given the hemodynamical compromise. We withdrew the wire and microcatheter from the proximal septal branch to allow perfusion, and the patient stabilized.

**Figure 7:**
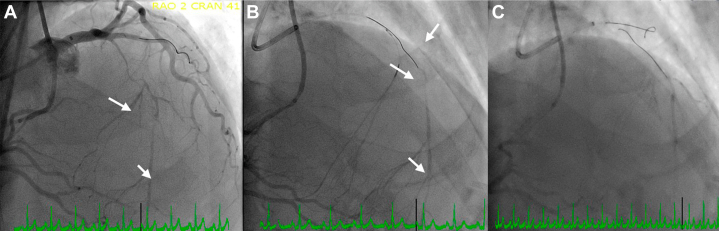
LAD CTO Percutaneous Coronary Intervention Initial Steps Left heart catheterization showing (A) dual TrapLiner catheters in place, upfront retrograde approach. The arrows indicate the septal branches. (B) We were unable to wire the true lumen in the distal left anterior descending artery after the first septal branch and therefore were unable to pass the septal branch using the retrograde microcatheter, (C) hence, we decided to wire the second septal branch. The arrows in B indicate the left anterior descending coronary artery and diagonal branch.

**Figure 8:**
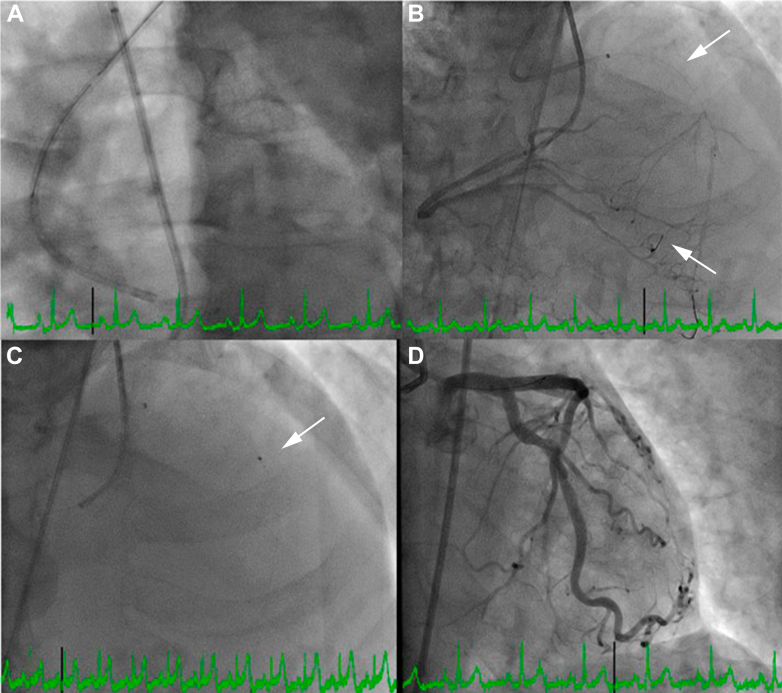
LAD CTO Percutaneous Coronary Intervention Final Steps Left heart catheterization showing (A) dual injection system. (B) FineCross catheter advanced to the second septal branch and BMW wire placed in the distal LAD; converted to antegrade approach. (C) An 0.9-mm laser atherectomy performed in the left anterior descending artery. (D) Final angiogram of the left anterior descending artery showing TIMI flow grade 3. The upper arrow in B indicates FineCross catheter and the lower one in indicates Suoh 03 wire in the distal septal branch. The arrow in C indicates tip of the laser atherectomy catheter.

We then approached the smaller distal septal branch. We crossed the septal branch and the LAD lesion with the Suoh wire (Video 1). Our microcatheter could not cross the distal septal branch, hence we could not externalize the wire. Antegrade, we had a FineCross microguide catheter extending through the antegrade TrapLiner catheter. We then inflated the TrapLiner trapping balloon and performed the tip-in technique on our retrograde Suoh wire into the antegrade FineCross catheter (Video 2). We then deflated the TrapLiner trapping balloon and advanced the FineCross into the mid-LAD over the Souh wire. The wire was then retracted into the distal septal branch, and a new BMW wire antegrade was wired down the LAD (Figure 7). Retrograde angiography confirmed wire location; therefore, we decided to work over the BMW wire at that point in time. We used 2.0-mm and 1.5-mm balloons but were unable to advance across the proximal-mid LAD. Laser atherectomy was then performed (Video 3) using 80 mJ/mm^2^ fluence and 80 Hz repetition rate, after which we performed intravascular ultrasound. Percutaneous transluminal coronary angioplasty was then performed with a 2.5-mm balloon. PCI was performed with a 2.75 × 23 mm DES in the mid LAD, overlapping with a 3.5 × 33 mm DES in the proximal LAD, with stent postdilation using a 2.75 mm NC balloon distally for the mid-LAD stent and a 3.75 mm NC balloon proximal and midstent. Angiography was performed showing a distal lesion seen after the stents. Therefore, a third stent was placed in the midsegment (after lesion preparation with a 2.5-mm NC balloon). The stented segments were all postdilated to high pressure. Final angiography showed TIMI flow grade 3, with no edge dissection noted and all stents well expanded (Figure 8). No branches were lost, and the patient was pain free at the end of the procedure.

The patient had an uncomplicated postprocedural hospital course and was discharged on dual antiplatelet therapy and goal-directed medical therapy for cardiomyopathy. He was doing well on clinic follow-up visits at 1 week and 1 month postdischarge, asymptomatic from a cardiac perspective.

## Discussion

Coronary CTOs are detected during coronary angiography and require complex treatment plans. Despite patients experiencing angina, the majority of patients with CTO remain unrevascularized given concerns about technical complexity and perceived high failure rates.^3^ There are different approaches to address a CTO lesion: antegrade, retrograde, or a combination of the two. The approach depends on several factors, including lesion length, lesion cap characteristics, and suitability of collaterals for retrograde approach.^3^ The main goal of these approaches is to achieve revascularization of the ischemic area while optimizing clinical outcomes and preserving the structural and functional integrity of critical side branches and outflow vessels.^3^

The use of laser in retrograde CTO-PCI has been explored as a technique to enhance procedural success in complex cases. Excimer laser coronary atherectomy (ELCA) is particularly useful in scenarios where lesions are balloon uncrossable or undilatable, which are common challenges in CTO-PCI. This is primarily using the photomechanical effect of a laser to allow it to cross lesions. A study by Karacsonyi et al^4^ demonstrated that compared to cases where laser was not used, laser use in balloon uncrossable and undilatable CTO lesions was associated with both higher technical success rate (91.5% vs 83.1%) and procedural success rate (88.9% vs 81.6%). This finding suggests that laser can be an effective adjunct in overcoming these specific intraprocedural obstacles.

Advancements in laser technology and techniques, such as the contrast infusion method, have improved the efficacy and safety of ELCA in these challenging cases.^5^ However, laser use in CTO-PCI is infrequent and almost not heard of in retrograde CTO-PCI. Case reports and series have further illustrated the practical application of laser in CTO-PCI, and more knowledge sharing is needed to bridge the gaps and further develop CTO-PCI algorithms. Shen et al^6^ reported successful recanalization of a calcified balloon-uncrossable CTO using a laser catheter, emphasizing its role in selected patients in whom conventional methods fail. Similarly, Sapontis et al^7^ described the use of ELCA in overcoming various intraprocedural obstacles, including balloon-resistant lesions and device resistance for in-stent restenosis. In our case, we used laser atherectomy as part of a retrograde CTO-PCI, which has it is own set of challenges.

The tip-in technique is beneficial in complex scenarios involving extreme vessel angulation, severe calcification, fragile collaterals, and challenging retrograde microcatheter crossing without externalization.^8^ It serves as a strategic advantage in retrograde CTO-PCI, providing a valuable and feasible alternative to conventional retrograde connection techniques, particularly when those techniques fail.^8^ The tip-in microcatheter technique with use of the TrapLiner guide extension catheter, used in the present case, is a unique technique that can help improve procedural success rates in retrograde CTO-PCI, particularly in uncrossable septal branches with difficulty externalizing the retrograde wire. This approach offers a strategic advantage by reducing potential damage to collateral channels and the ostium of the donor artery, potentially leading to a reduction in complication rates.^9^

## Conclusions

While the retrograde approach is critical for achieving high success rates in complex CTO cases, the use of laser in specific scenarios such as balloon uncrossable and undilatable lesions can significantly enhance procedural success. This suggests that laser atherectomy can be a valuable adjunct in retrograde CTO-PCI, particularly in overcoming specific intraprocedural challenges. Using advanced techniques including tip-in maneuvers and equipment such as the TrapLiner can help us succeed in complex cases that may otherwise fail.

## Funding Support and Author Disclosure

The authors have reported that they have no relationships relevant to the contents of this paper to disclose.

## Appendix

Video 1 The Steps Used to Cross the Smaller Distal Septal Branch Into the LAD Lesion With a Suoh 03 Wire

Video 2 Tip-In of a Retrograde Suoh 03 Wire Into the Antegrade FineCross Microcatheter, Then Advancing the Microcatheter Into the Mid-LAD Over the Suoh Wire

Video 3 Laser Atherectomy in the Proximal-Mid LAD Using an 0.9-mm Excimer Laser Coronary Atherectomy System
